# Clinical Lectures on Diseases of the Eye

**Published:** 1864-08

**Authors:** E. L. Holmes

**Affiliations:** Chicago, Lecturer on Diseases of the Eye and Ear in Rush Medical College, and Surgeon of the Chicago Charitable Eye and Ear Infirmary


### CLINICAL LECTURES ON DISEASES OF THE EYE.

By E. L HOLMES, M. D., of Chicago,
*Lecturer on Diseases of the Eye and Ear in Rush Medical College, and Surgeon of the Chicago Charitable Eye and Ear Infirmary.*

### CHRONIC CONJUNCTIVITIS—GRANULATED LIDS.

Gentlemen:—The form of disease to which I ask your attention to-day is one of the most common, and in some respects the most obstinate, affections of the eye, which you will have occasion to treat.

It is found in all classes of society, but more frequently among the poor. At the West there is scarcely a malady which causes more physical and mental suffering than this. It is, therefore, worthy your especial attention.

The symptoms of this affection do not need a long description. A single inspection of the palpebral conjunctiva is sufficient to form a diagnosis.

There are two principal forms of granulations—a simple hypertrophy of the papillæ, as also of the mucous membrane itself, and a form, characterized by the presence of yellowish white gelatinous elevations, scattered upon the hypertrophied conjunctiva. These two forms are very often found existing in different degrees in the same eye. Occasionally a small portion of the lids nearest the external and internal angles is alone affected. The semilunar fold, with the caruncla and the duplicature, are perhaps the portions most liable to take on this condition.

There is usually an increased secretion of tears, mucus, and from the meibomian glands, which is especially observable after sleep. The lids are more or less tumified and tend to keep partially closed. The eyes become readily fatigued in reading or writing, especially by artificial light. Vision is, to a certain extent, impaired by the increased amount of mucus spread over the cornea. The vessels of the sclerotical conjunctiva are apt to be congested, and to encroach upon the cornea, especially its upper portion. In time, from the constant rubbing of the roughened lids, the cornea becomes ulcerated and so covered with vessels as to destroy vision. It is wonderful, however, to mark the differences in the effects of this disease in different individuals. While one patient, with a very slight degree of granulated lids, will experience so much irritation and disease of the cornea as to render him almost helpless, another may have, for a long time, enormous masses of granulations upon the conjunctiva, and yet the cornea may remain almost perfectly clear. Many of the differences which you will observe in different cases are inexplicable.

The course of the disease, after it is once established, and not modified by treatment, and the manner in which it proves destructive to the vision, by secondary disease of the cornea, have already been described. If this secondary disease does not supervene from the direct effects of the granular conjunctivitis, it is almost certain to follow from the effects produced upon the lids. You have already seen several cases illustrating the manner in which the lids are permanently injured by this disease. The conjunctiva and tarsal cartilages eventually become atrophied and covered with cicatrices; the tissues of the lids become so contracted as to either change the direction of the cilia by turning the whole edge of the lids against the globe, as in entropion, or by changing the relative position of the follicles of the cilia themselves, as in trichiasis. In this complication the lids may remain, for a period, quite normal in appearance. The hardened contractions and cicatrices of the conjunctiva, with the misdirected cilia, rub upon the cornea and almost inevitably produce its destruction.

The chief cause of Trachoma is neglected or mal-treated conjunctivitis. At the West that form of inflammation termed catarrhal, is the most prevalent cause. Muco-purulent and purulent conjunctivitis, if not speedily arrested, are almost certain to produce "granulations." With some patients there seems to be no apparent cause; without change of external circumstances, without active inflammation or marked congestion, they complain of trifling irritation of the eyes, especially in reading. On an examination of the lids, the palpebral conjunctiva will be found covered with fine elevated points of granulations and enlarged vessels. I have found this form of disease not unfrequent, and although it is sometimes difficult to detect any evident causes, it is often the result of too much work with the eyes, and of constant exposure to slight causes of irritation, as dust, wind and smoke.

In all cases of chronic conjunctivitis, with granulations or hypertrophy following acute inflammation, you should be exceedingly guarded in giving a prognosis. This can not be called a disease, which tends to recovery by the simple efforts of nature. In very young children there is perhaps more hope of total recovery without treatment than in adults. Patients with good general health, and surrounded with favorable circumstances, are in less danger than those who do not possess these advantages. That class of patients to which I have alluded, with simple uncomplicated granulations, without previous symptoms, are usually readily relieved by suitable treatment.

But with patients as they are generally found, who, from neglect, ill-treatment and exposure, have suffered from acute and chronic conjunctivitis, till the mucous membrane has become covered with granulations, the tissues atrophied and the cornea ulcerated, I consider the prognosis in regard to *perfect* vision as exceedingly grave. Unfortunately these patients are principally among the poor classes of society; they are often deprived of suitable food, and by the ordinary performance of their duties are exposed to the active causes of disease. Such patients, when suffering from this disease, seldom continue treatment after they can resume their labors, even if they are not cured. The disease soon reappears with its former violence; treatment is again sought with the same results. Relapse follows relapse till vision is permanently injured.

I think any one who has watched the treatment of this class of cases in the European clinics, and also in the Eye Infirmaries of this country, will be convinced that trachoma as usually observed, is a very serious disease. In a large proportion of cases just described I believe it is incurable, in the strict meaning of this word, for there will remain for years, even if vision is almost normal, a peculiar tendency to congestion from slight causes; upon examining the conjunctiva it will be found to present an appearance differing from that of health.

In reference to treatment, I have little to say in comparison with what has been written upon this part of our subject. I can only offer a few suggestions, referring you at the same time to the chapters in our best text-books to convince you that the combined experience of the world has failed to discover a plan of treatment which has removed "granulated lids," from the list of the opprobria of medicine. Still you must not suppose we can do nothing. By patience and judicious use of suitable remedies you will sometimes absolutely cure severe cases of this disease, and very often so far relieve pain and discomfort as to enable patients to pursue their ordinary occupations.

As regards the patients themselves, there are three distinct classes, each requiring in some respects a different course of treatment. The first comprises healthy, vigorous individuals, in whom the disease is, perhaps, purely local. The second may include those who are of a sound constitution, but who, from their disease, pain, anxiety, want of exercise, pure air and attention to the ordinary rules of health, have been reduced in strength and vitality. The third class includes those who suffer from a bad diathesis, the scrofulous and the syphilitic. The treatment of the first class is almost entirely local; in the second class, the health must be restored by placing the patient, as far as possible, in such relations as will improve his spirits and strength. The use of tonics and restoratives are also indicated. The third class of patients require constitutional treatment—usually termed alterative—with strict attention to everything that can effect the general health.

Patients should be removed from the influence of whatever can irritate the eyes, as dust, smoke, and too brilliant light. Absolute rest from the use of the eyes is demanded. Nutritious diet, but not in too large quantities, is required, and in ordinary circumstances patients should not be permitted to sleep through the day, especially after eating, since the eyes tend to passive congestion during sleep.

Collections of mucus and other secretions around the eyes should be carefully removed by gentle sponging with tepid water. At night the edges of the lids should be covered with some form of simple ointment to prevent the accumulation of hardened secretions and consequent adherence of the lids.

The direct treatment of the disease consists in the application of caustic astringents and in the removal of the granulations by means of the knife or scissors. The latter plan is only indicated in cases of elevated, wart-like granulations, and when adopted, great care should be taken not to include the true conjunctiva, the destruction of which is liable to produce uneven cicatrices.

Skill in the use of local caustics depends almost entirely upon the selection of the proper strength. Although there are several of these remedies which may be used with benefit in different cases, after trying nearly all of them, I now de-depend in most cases, upon nitrate of silver, crystal sulphate of copper, and liquor plumbi subacetatis, principally, however, upon the two former.

It is difficult to describe the various conditions of the conjunctiva which you will observe in different cases of this disease. And yet it is important for you to learn what cases usually require strong, and what weak solutions of caustic. I shall, therefore, be obliged to depend upon the cases which I shall, from time to time, bring before you, to illustrate this subject, and shall take particular pains to point out the peculiarities of each case with the strength of the remedy best adapted to it.

I would advise you to use the solid nitrate of silver very seldom. This caustic, reduced in strength one-half, two-thirds or three-fourths, by means of nitrate of potash melted into sticks with it, may be used with benefit, although I almost invariably apply the remedy in solution, with a brush, as already described. Strong solutions should not be dropped into the eye, since they can not in this way be applied equally over the diseased membrane, and the secretions from the eye destroy their full action. It is well for you to commence with weak solutions, gradually increasing their strength according to the effects produced.

In acute conjunctivitis I am inclined to believe practitioners usually apply caustics too weak; in chronic inflammation too strong. If you pursue the course I have just advised, there will be little danger of doing injury. The sulphate of copper if in the form of a smooth crystal, is a most valuable remedy in a large number of cases. As a rule, you will find one application daily of the above mentioned remedies sufficient. Trial alone will teach you whether the treatment should be repeated daily or three times a week.

Whenever the cornea is affected with granulations or vascularity in connection with trachoma, the latter disease must generally be relieved before the cornea will improve.

